# Translating current basic research into future therapies for neurofibromatosis type 1

**DOI:** 10.1038/s41416-020-0903-x

**Published:** 2020-05-22

**Authors:** Jean-Philippe Brosseau, Chung-Ping Liao, Lu Q. Le

**Affiliations:** 1 Department of Dermatology, University of Texas Southwestern Medical Center at Dallas, Dallas, TX 75390-9069 USA; 2 Simmons Comprehensive Cancer Center, University of Texas Southwestern Medical Center at Dallas, Dallas, TX 75390-9069 USA; 3 UTSW Comprehensive Neurofibromatosis Clinic, University of Texas Southwestern Medical Center at Dallas, Dallas, TX 75390-9069 USA; 4 Hamon Center for Regenerative Science and Medicine, University of Texas Southwestern Medical Center at Dallas, Dallas, TX 75390-9069 USA; 5 Present Address: Department of Biochemistry and Functional Genomics, University of Sherbrooke, Sherbrooke, QC J1E 4K8 Canada

**Keywords:** Cancer genetics, Tumour-suppressor proteins

## Abstract

Neurofibromatosis type 1 (NF1) is a hereditary tumour syndrome that predisposes to benign and malignant tumours originating from neural crest cells. Biallelic inactivation of the tumour-suppressor gene *NF1* in glial cells in the skin, along a nerve plexus or in the brain results in the development of benign tumours: cutaneous neurofibroma, plexiform neurofibroma and glioma, respectively. Despite more than 40 years of research, only one medication was recently approved for treatment of plexiform neurofibroma and no drugs have been specifically approved for the management of other tumours. Work carried out over the past several years indicates that inhibiting different cellular signalling pathways (such as Hippo, Janus kinase/signal transducer and activator of transcription, mitogen-activated protein kinase and those mediated by sex hormones) in tumour cells or targeting cells in the microenvironment (nerve cells, macrophages, mast cells and T cells) might benefit NF1 patients. In this review, we outline previous strategies aimed at targeting these signalling pathways or cells in the microenvironment, agents that are currently in clinical trials, and the latest advances in basic research that could culminate in the development of novel therapeutics for patients with NF1.

## Background

Neurofibromatosis type 1 (NF1), first characterised in detail by the German pathologist Friedrich von Recklinghausen in 1882, is an autosomal-dominant genetic disorder that results from biallelic inactivation of the tumour-suppressor gene *NF1*. The *NF1* gene encodes neurofibromin, a GTPase-activating protein (GAP) that negatively regulates the oncoprotein RAS. About half of all cases of the disorder involve de novo *NF1* mutations, which can be passed on to successive generations. The penetrance is almost 100%, but the expressivity varies greatly, even between twins. Indeed, even though 99% of NF1 patients meet the diagnostic criteria (Box 1)^1^ by the age of 20 years,^2^ the manifestations are unpredictable even within the same family harbouring an identical mutation. This observation has given rise to the spatiotemporal hypothesis, which proposes that, in addition to the nature of the *NF1* mutation and additional genetic/epigenetic differences, the timing and the exact cell population that is undergoing loss of the second functional copy of *NF1* are key factors that explain the overall phenotype.^3^

Clinically, NF1 affects 1 in every 3000 new-borns regardless of gender or race. This multisystem disorder has diverse manifestations including neurocognitive, skeletal and cardiovascular abnormalities, and is also classified as a neurocutaneous syndrome to highlight its manifestations primarily in the nervous system and the skin as a result of lesions affecting neural-crest-derived tissues such as Schwann cells and melanocytes.^4^

NF1 patients are predisposed to develop multiple types of benign and malignant tumour, including neurofibromas, malignant peripheral nerve sheath tumours (MPNSTs) and optic pathway gliomas (OPGs). Gastrointestinal stromal tumours, breast cancer, juvenile myelomonocytic leukaemia, rhabdomyosarcoma and pheochromocytoma^5^ are also associated with NF1; however, these neoplasms occur at a much lower frequency and will not be discussed in this review. Neurofibromas are benign tumours of the nerve sheath that are composed of neoplastic Schwann cells and non-neoplastic fibroblasts, mast cells, macrophages, endothelial cells, nerves and other cell types as well as abundant collagen deposition. Neurofibromas develop in the peripheral nervous system, and can be generally grouped into two main subtypes—cutaneous neurofibromas (cNFs) and plexiform neurofibromas (pNFs)—by their locations. cNFs, which occur in nearly all NF1 patients and are one of the defining features of the disorder, are discrete masses associated with cutaneous nerves, the development of which coincides with puberty.^6^ However, the clinical manifestations in cNF can be highly variable depending on individuals; some patients develop only a few visible tumours, whereas the entire body is covered in other cases. By contrast, pNFs arise from multiple nerve fascicles internally and infiltrate the surrounding soft tissue. Most pNFs are believed to be congenital and are more likely to grow during childhood. In the cases of cNF and pNF, no additional pathogenic mutation is recurrently found in neurofibroma except the one located in *NF1*.^7,8^ Clinical management largely comprises surgical resection, and no effective pharmacological agent is yet available for effective treatment or cure, although some agents have shown promising results in preclinical or clinical trials, and will be discussed in detail in this review.

A wide range of animal models (Box 2) has been developed to study the intracellular signalling pathways as well as extracellular signals that regulate tumour development in NF1. Tumorigenesis is a multi-step process that progressively transforms a normal tissue into a benign one (e.g. cNF, pNF, OPG) and potentially subsequently into a malignant one (e.g. MPNST). In the context of NF1-related tumours, it is unclear whether all NF1-associated MPNSTs originate from a pre-existing pNF. However, a pNF can progress into an atypical neurofibromatous neoplasm of uncertain biological potential (ANNUBP)^9^ typically as a result of a subsequent genetic mutation in *CDKN2A*^10,11^ and, upon further genetic insult to additional tumour-suppressor genes, such as *TP53*, *PTEN* or members of the PRC2 chromatin-remodelling complex,^12^ an ANNUBP or a pNF can transform into a MPNST. The lifetime risk of developing MPNSTs is 8–13% in all patients with NF1,^13^ although this risk is higher in patients with pNFs. MPNSTs are life-threatening, highly morbid, and show a high propensity for metastasising. Surgical resection of MPNSTs is challenging because these tumours are commonly inaccessible within the body or they infiltrate vital nerves—hence the urgent need for effective therapeutic treatment.

NF1 patients can also develop OPGs. These low-grade brain tumours affect young children, with most being diagnosed before the age of 5 years old. Although OPGs rarely cause death, they can lead to vision loss and even blindness in many patients.^14^ However, the mechanism of vision loss in OPGs is still unclear. First-line chemotherapy, using vincristine and carboplatin, for example, is usually effective at stabilising the disease^15^ but it is still challenging to predict who will benefit from this treatment as well as to decide who actually needs treatment. The microenvironment of OPGs is very heterogeneous and plays an essential role in tumorigenesis as in neurofibromas. OPGs are composed of astrocytes, oligodendrocytes, neurons and microglia (the nervous system equivalent of macrophages).^16^ Several studies have demonstrated that OPG tumour cell proliferation is largely dependent on the microglia,^17–19^ which secrete chemokine ligands to recruit more microglia and to induce the proliferation of astroglial tumour cells.^20^ In addition, microglia can cause neurotoxicity (e.g. by releasing reactive oxygen species), which can damage optic nerve axons.^21^

The focus of this review is to outline therapeutic strategies that have previously been developed and tested, agents that are currently being tested in clinical trials and future therapies that extrapolate from the latest published basic research in the laboratory for the treatment of the most common NF1-related tumours, including cNFs, pNFs, MPNSTs and OPGs.

Box 1: The diagnostic criteria for NF1 Currently, the diagnosis of NF1 requires two or more of the following criteria to be fulfilled:

1. The appearance of six or more café-au-lait spots that measure more than 5 mm in children or more than 15 mm across in adolescents and adults.

2. Freckling in the area of the axilla or the groin.

3. Two or more Lisch nodules (melanocytic hamartomas) on the iris of the eye.

4. Two or more cutaneous neurofibromas (cNFs) or one plexiform neurofibroma (pNF).

5. An optic pathway glioma (OPG).

6. Skeletal abnormalities (thinning of the long bones, sphenoid wing dysplasia).

7. A parent, sibling or child with NF1.

Box 2: Animal models for studying NF1 The first neurofibroma mouse model was established by depletion of the *Nf1* gene in *Krox20* lineage Schwann cells.^116^ Subsequently, similar neurofibroma mouse models have been successfully created by ablation of *Nf1* under various Schwann cell promotor Cre lines, such as *Dhh*,^117^ *P0A*^118^ and *Plp*.^99^ Strikingly, these mice exclusively developed pNFs as no strong evidence of cNFs was reported. It took almost two decades to identify the specific subpopulation of neural crest cells that migrate into the subepidermal region in the skin and eventually differentiate into glial cells. Ablation of *Nf1* in this cell population gives rises to both cNF and pNF, mirroring the clinical scenario.^3,119^

In addition to mice, independent research groups have successfully developed NF1 pig models.^120,121^ Miniswine with targeted deletion of *Nf1* undergo loss of heterozygosity in neural-crest-derived cells, recapitulating human-like cNFs^120,121^ and OPGs.^120^ It remains to be demonstrated whether these models faithfully develop pNFs and MPNSTs.

A wide range of mouse models are currently used to recapitulate human MPNSTs. Spontaneous transition from pNF to MPNST in mouse models is relatively rare but has been observed.^11,81,96,107^ In the majority of these MPNST mouse models, functional deletion of NF1 and additional relevant tumour-suppressor genes directly leads to the development of MPNSTs without apparent neurofibroma formation.^118,122–125^ Xenograft models are attractive and widely used for preclinical studies of MPNSTs.^126^

Although there are limited insights from human clinical OPG samples (because biopsies are rarely performed), OPG has been fully recapitulated by several mouse models by ablation of the *Nf1* gene in glial progenitor cells^16,127,128^ residing in the third ventricle.^129^

## Therapeutic strategies developed and tested in the past

**Fig. 1:**
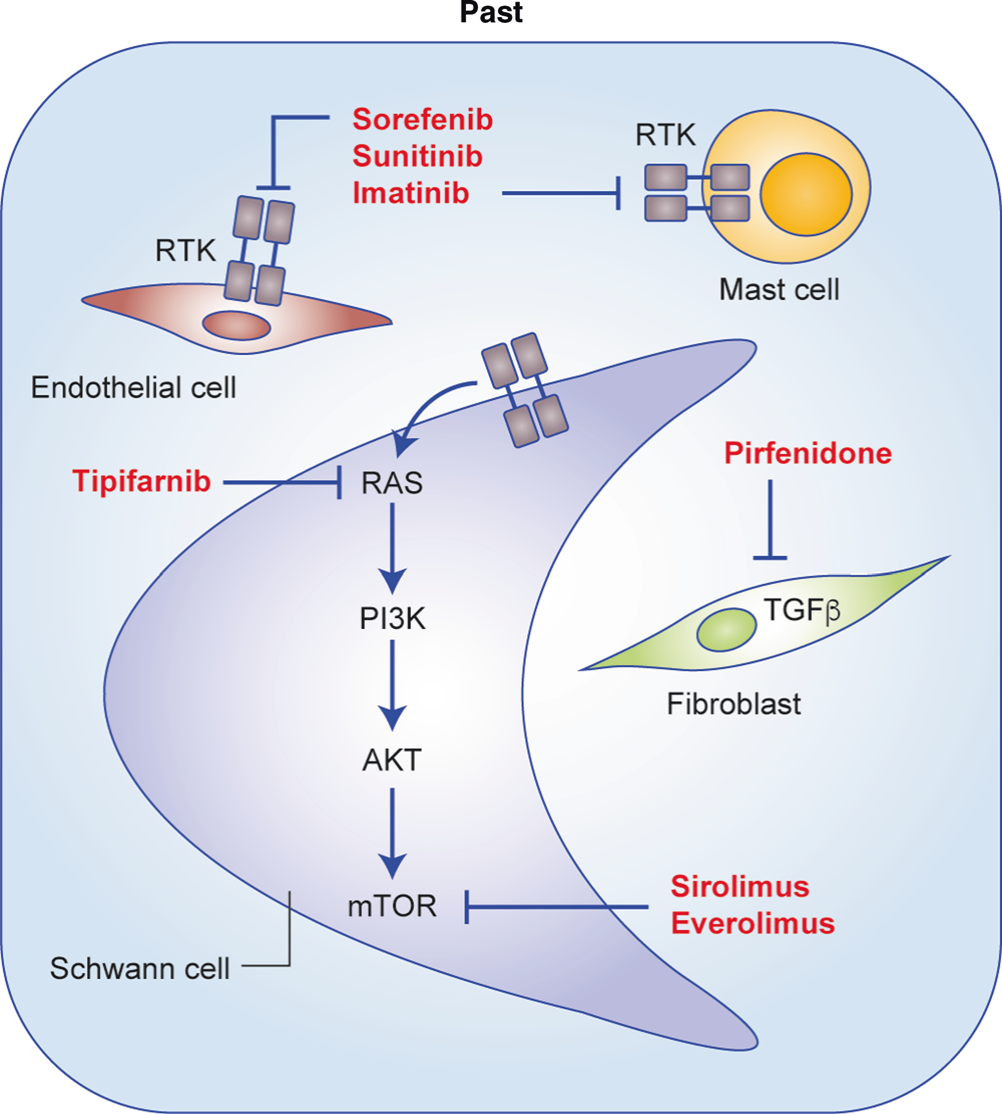
Therapeutic strategies developed and tested in the past. Overview of previously trialled therapies for the most common types of tumour associated with NF1. Therapeutic agents include the farnesyltransferase inhibitor ipifarnib, inhibitors of receptor tyrosine kinases (RTKs) upstream of RAS (imatinib, sunitinib, sorafenib), inhibitors of components of the pathway downstream of RAS (mTOR inhibitors sirolimus, everolimus) and anti-fibrotic agents such as pirfenidone.

Significant progress has been made by the neurofibromatosis research community, including the genetic cause of NF1, the cellular function of the neurofibromin protein, and the identification of cellular components that contribute to the tumours developed in NF1. This knowledge has been translated into clinical trials of putative therapeutic targets in an attempt to develop new treatments for NF1 (Fig. 1).

### Upstream of Ras: receptor tyrosine kinase inhibitors

The best characterised biochemical function of NF1 is the inhibition of Ras signalling mediated by its GAP domain. Ras (KRAS, NRAS and HRAS) is the most frequently mutated oncogene in human cancer^22^ but is notoriously ‘undruggable’.^23^ Indeed, directly targeting Ras using, among other agents, the farnesyltransferase inhibitor tipifarnib has been extremely challenging in general^24^ and was also demonstrated to be ineffective in the treatment of pNF^25^ (NCT00021541). As Ras itself is not mutated in NF1, an alternative strategy has been to target the upstream activators of Ras—receptor tyrosine kinases (RTKs).

Epidermal growth factor receptor (EGFR) is an RTK that is abundantly expressed in neurofibroma and MPNST cell lines, despite not normally being expressed in Schwann cells.^26^ Indeed, expressing EGFR in Schwann cells in transgenic mice leads to the formation of neurofibroma-like lesions (although at a very low penetrance), whereas an inactivating EGFR mutation decreases its tumorigenic potential in a mouse model of MPNST.^27^ However, in humans, the EGFR inhibitor erlotinib largely failed to inhibit tumour growth in a Phase 2 trial of patients with advanced stage MPNST^28^ (NCT00068367). It remains to be determined if erlotinib or another potent EGFR inhibitor would be clinically effective in the context of pNF, cNF or OPG, and whether or not patient stratification based on high level of EGFR positivity would be a better approach.

Platelet-derived growth factor receptor (PDGFR) is another RTK that is overexpressed in Schwann cells derived from neurofibromas^29^ and MPNSTs,^30^ and the expression levels of the c-met RTK proto-oncogene product and its ligand hepatocyte growth factor (HGF) correlate with disease progression.^31^ However, despite promising results from preclinical studies, including the targeting of mast cells through inhibition of the c-Kit RTK,^32^ when inhibitors targeting various relevant RTKs were tested in clinical trials using sorafenib for patients with OPG^33^ (NCT01338857) or imatinib for pNF^34^ (NCT01673009) and MPNST^35^ (NCT00031915), disappointingly, they largely lacked efficacy at reducing tumour burden.

Neurofibroma is highly vascularised,^36^ and the vascular endothelial growth factor (VEGF) ligand is known to be expressed in cNF^37^ and at even higher levels in MPNST, where it correlates with poor patient prognosis.^38^ A small molecule inhibitor targeting the RTK VEGF receptor 2 (VEGFR2) decreased angiogenesis and proliferation by over 50% in a tumour explant xenograft model^39^ but, unfortunately, in clinical trials, targeting VEGF-A using the monoclonal antibody ranibizumab was not effective for cNF (NCT00657202), and targeting VEGFR through the RTK inhibitor sorafenib was not effective for MPNST^40^ (NCT00245102). However, as sorafenib has multiple targets, it is not appropriate to simply imply that failure to inhibit VEGFR is the main reason for this result. On a positive note, cabozantinib, a small molecule inhibitor of the RTKs VEGFR2, c-MET, AXL and RET, was reported at the 2018 Joint Global Neurofibromatosis Conference to have some efficacy against pNF (NCT02101736). Furthermore, when treated with bevacizumab, a monoclonal antibody targeting VEGF-A, some children with OPG have been reported to respond positively, as indirectly judged by their improved vision.^41^

### Downstream of Ras: mammalian target of rapamycin inhibitor

The mammalian target of rapamycin (mTOR) pathway is a central survival pathway downstream of Ras. Inhibition of mTOR is effective in the context of tuberous sclerosis, another neurocutaneous disease, prompting investigation of pharmacological inhibition of the mTOR pathway for NF1. However, although this approach decreased tumour cell proliferation and, consequently, tumour volume in an NF1 mouse model,^42,43^ mTOR inhibitors were ineffective against pNF in one clinical trial^44^ but prolonged progression-free survival, albeit modestly, in another trial^45^ (NCT00634270). A combination trial of mTOR inhibition plus bevacizumab was also ineffective for MPNST^46^ (NCT01661283). The mTOR inhibitor everolimus did, however, show promising early results in a pilot trial for cNF^47^ (NCT02332902) and is currently being tested in a clinical trial in patients with OPG (NCT01158651).

### Anti-fibrotic agents

Von Recklinghausen coined the term ‘neurofibromatosis’ to highlight the abundance of nerve cells and fibrosis in neurofibromas. A hallmark of these tumours is the presence of collagen, which can comprise up to 50% of the dry weight of a tumour.^48^ As fibroblasts are collagen-producing cells and, by definition, fibrosis is an excess of collagen deposition, it has been hypothesised that neurofibroma constitutes ‘nerve fibrosis’. In support of this model, transforming growth factor β (TGF-β), one of the key signalling molecules involved in fibrosis, is secreted by mast cells upon stimulation by neoplastic *Nf1*^–/–^ Schwann cells.^49^ This hypothesis led to the testing of the broad anti-fibrotic pirfenidone, which has proven efficacy for idiopathic pulmonary fibrosis, in Phase 2 trials in NF1 patients with advanced pNF but, unfortunately, no benefit of pirfenidone was reported^50^ (NCT00076102). However, it is not clear whether the trial was unsuccessful because pirfenidone was not effective at reducing collagen deposition or because collagen type I is not an effective drug target in pNF. Further investigation is needed to definitively address the contribution of collagen to NF1-related tumour development.

## Current therapeutics strategies under development

**Fig. 2:**
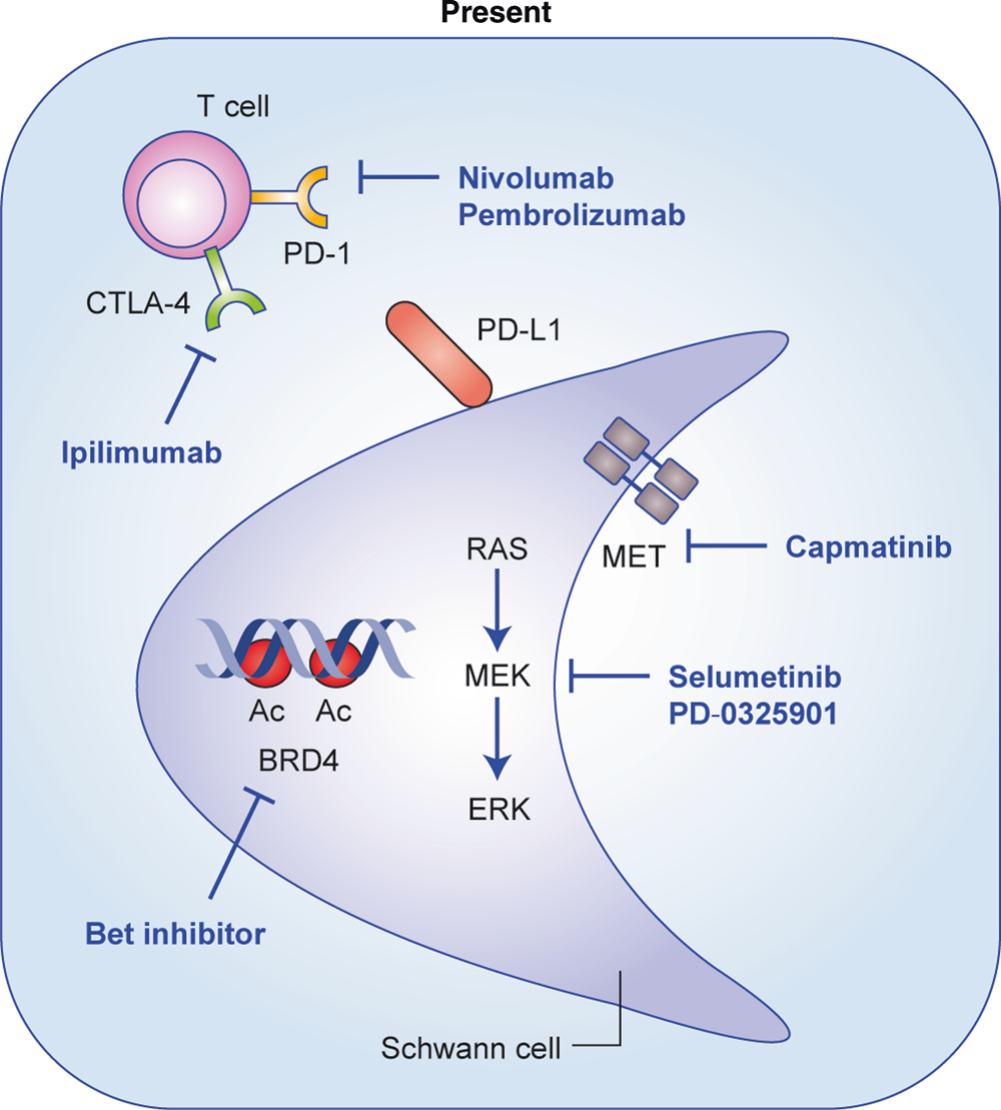
Current therapeutics strategies under development. Overview of current therapies under investigation for the most common types of tumour associated with NF1, including inhibitors of receptor tyrosine kinases (RTKs) upstream of RAS (capmatinib), components of the pathway downstream of RAS (MEK inhibitors), immune-checkpoint inhibitors (ipilimumab, nivolumab, pembrolizumab) and BET inhibitors.

The existence of mouse models in the NF1 arena (Box 2) has provided unprecedented opportunities to derive important insights into the biology of the disease as well as preclinical models that guide the development of effective therapies for NF1 (Fig. 2)

### Downstream of Ras: mitogen-activated protein kinase kinase inhibitors

Downstream of Ras activation, the extracellular-signal regulated kinase/mitogen-activated protein kinase (ERK/MAPK) signalling pathway relays proliferation and cell-cycle entry signals. This pathway is hyperactivated in NF1-related tumours, and inhibition of MAPK kinase (MEK) was effective at controlling tumour size in a mouse model of NF1.^51,52^ Unlike other therapeutic strategies tested so far, MEK inhibition (using selumetinib) has yielded unprecedented results in the clinic for pNF.^53^ In fact, almost all selumetinib-treated patients showed no tumour progression while being treated with the drug, with 72% showing a partial response and 24% showing stable disease in the SPRINT trial (NCT01362803). Based on these promising results, the FDA recently approved selumetinib for children with symptomatic, progressive and inoperable pNFs.  A small cohort of children with OPG were similarly responsive to MEK inhibition (NCT01089101)^54^ but equivalent strategies in the malignant context (in MPNST) have so far shown only moderate efficacy in mouse models.^55^ Of note, it has been reported that the RTK MET is activated in some MPNSTs and that a mouse model recapitulating MET activation in MPNST is sensitive to MET inhibition;^56^ it would therefore be interesting to test a variety of MEK combination therapies such as dual MET–MEK inhibition in the context of MPNSTs.^56^

### Inhibitors of bromodomain-containing protein 4

A comparative transcriptome analysis of mouse MPNSTs and their cells of origin revealed that the gene encoding bromodomain-containing protein 4 *(Brd4)* is one of the most highly upregulated genes in MPNSTs.^57^ BRD4 is a chromatin regulator that contains two bromodomains and an extended terminal domain characteristic of the BET family of bromodomain proteins. It binds to acetylated histone (H3K27Ac) and facilitates the expression of mitotic genes. Its downregulation or inhibition through the selective small inhibitor JQ-1 drastically decreases the growth of many types of cancer cell,^58^ including MPNST cells in vitro and in vivo.^57^ As many MPNSTs in NF1 patients have mutations in components of the PRC2 complex, which negatively regulates gene expression through histone methylation (H3K27Me) resulting in histone acetylation (H3K27Ac), inhibiting BRD4 might be particularly effective.^59^ This observation prompted the launch of a Phase 2 clinical trial of a small molecule inhibitor of BET domain proteins for MPNST, but the study was discontinued due to a lack of participants (NCT02986919). However, another study of a BET inhibitor plus a MEK inhibitor plus an inhibitor of programmed death-ligand (PD-L1) (see below) for MPNST is being carried out by the NF Clinical Trials Consortium.

### T cells

The discovery that cancer can escape the cytotoxic capacity of T cells through checkpoint strategies has great potential to revolutionise the field of cancer therapeutics but, despite the clinical success of immune checkpoint inhibitors,^60,61^ T cells have received little attention in the context of NF1. Immune profiling of neurofibroma and MPNSTs indicates considerable variation in the extent of infiltration of CD8^+^ T cells as well as in the expression level of the T-cell inhibitory ligand PD-L1;^62,63^ 50% of gliomas contain a high number of T cells.^64^ Although the influence of T cells on neurofibroma formation has not been directly evaluated in NF1-related tumours, these cells are emerging as essential components of the stroma that support tumour growth.^65,66^ Accordingly, clinical trials targeting T cells using a combination of antibodies against the receptor for PD-L1 (PD-1) and cytotoxic T-lymphocyte-associated protein 4 (CTLA4, another immune checkpoint protein) are currently ongoing (e.g. NCT02834013) in a Phase 2 trial for patients with rare tumours (including NF1-related tumours), although reliable markers predicting the response to immunotherapies remain to be identified in NF1 preclinical models.^67^

Immunocompromised (T-cell-deficient) mouse models are commonly used in cancer biology to perform xenograft studies. Intriguingly, malignant glioma cells successfully graft in immunocompromised mice whereas benign glioma cells fail to engraft.^65^ Pan and colleagues^65^ discovered that the cause of the failure of benign cells to engraft is due to impaired microglia function, including reduced expression of the chemokine receptor *Ccr2* and its ligand *Ccl5* in the immunocompromised mice; accordingly, mice deficient in *Ccl5* phenocopy T-cell-deficiency, as gliomas also fail to engraft in *Ccl5* knockout mice. Importantly, T cell exposure restores Ccr2 and Ccl5 expression in athymic microglia.^65^ These observations suggest that T cells support benign glioma growth but impair malignant transformation. It is tempting to speculate that a similar mechanism occurs in humans, because human MPNST cells can be grown in athymic mice^57^ but a xenograft model with human neurofibroma cells has not so far been reported, indicating that dampening T-cell function can restrain benign tumour development whereas enhancing T-cell function, on the other hand, can be used to prevent malignant transformation.

## Advances in basic research that could lead to the generation of novel therapeutics

**Fig. 3:**
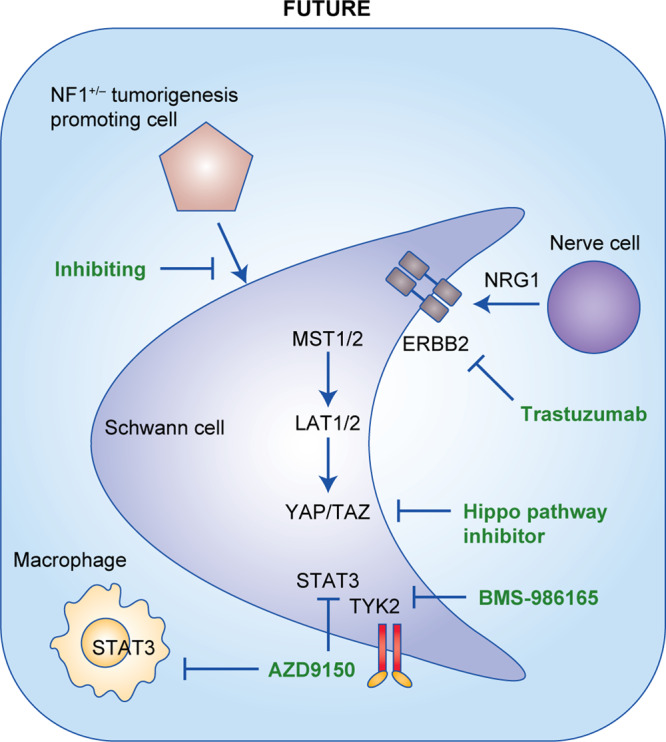
Advances in basic research that could lead to the generation of novel therapeutics. Overview of the potential future therapies for the most common types of tumour associated with NF1, including targeting the Hippo pathway, JAK/STAT signalling pathway and oestrogen signalling as well as components of the NF1^+/−^ tumour microenvironment (fibroblasts, nerve cells, macrophages and other immune cells).

Recent advances in our understanding of tumour molecular and cellular pathogenesis will provide opportunities to develop effective treatments in the future for NF1-associated neoplasms (Fig. 3).

### The Hippo pathway

As a central regulator of cell growth and size, it is not surprising that the Hippo pathway is involved in a number of tumour types, including NF2-associated tumours.^68^ The NF2 disease, characterised by highly penetrant tumours of the vestibular nerve called vestibular schwannomas, is caused by biallelic inactivation of the *NF2* gene. The protein encoded by the *NF2* gene, Merlin, functions as an upstream initiator of the core kinase signalling cascade involving MST1/2, LATS1/2 and the transcription factors YAP and TAZ. Interestingly, biallelic inactivation of the *NF2* gene leads to some features of NF1, such as cNF.^69,70^ Indeed, in genetically engineered mouse models of cNF and MPNST, the Hippo pathway acts as a modifier^3^ and a driver,^71^ respectively, suggesting that selectively dampening the Hippo pathway using inhibitors could be an attractive therapeutic approach, and several novel small molecule YAP inhibitors are currently in preclinical development.^72^ However, the fraction of NF1 patients that have an aberrant Hippo pathway and that, hence, might be sensitive to inhibition of the Hippo pathway, remains to be established.^3,73–75^

### The Janus kinase/signal transducer and activator of transcription pathway

Signalling through the Janus kinase (JAK)/signal transducer and activator of transcription (STAT) pathway is known to contribute to tumorigenesis in numerous ways.^76^ Using a high-throughput chemical library screen, Banerjee and colleagues discovered that STAT3 is hyperactivated in human glioma and MPNST cell lines.^77^ As EGFR is one of the tyrosine kinases that mediates STAT3 phosphorylation^78^ and is expressed in most human MPNSTs,^26^ researchers investigated, both genetically and pharmacologically, the contribution of STAT3 signalling in mouse models of pNF and MPNST.^79–81^ In brief, interfering with the STAT3 pathway delayed neurofibroma initiation^80^ as well as progression to MPNST,^81^ but had far less impact on tumour maintenance. The results of these preclinical studies suggest that inhibiting STAT3 might be of benefit to NF1 patients by preventing/delaying the development of pNFs. The safety and overall effect(s) of long-term STAT3 inhibition in humans are currently unknown, but data from early clinical trials indicate that antisense oligonucleotides that target STAT3 mRNA are well tolerated in the short term.^82^

The tyrosine kinase TYK2, also a member of the JAK family (in addition to JAK1–3), is emerging as an attractive target in the context of MPNST. This protein associates with the cytoplasmic domain of type I and type II cytokine receptors to ultimately activate downstream oncogenic signals.^76^ Overexpression of TYK2 was found in approximately 60% of MPNST cases but not in normal peripheral nerve or benign neurofibromas.^83^ In vitro, knockdown of TYK2 in murine and human MPNST cells significantly increased cell death, while knockdown in vivo led to a decreased tumour volume in a subcutaneous mouse model, supporting a role for TYK2 in driving MPNSTs.^84^ Importantly, orally available TYK2 inhibitors have been developed for the treatment of autoimmune disorders, and have so far proven to be clinically safe.^85,86^ It remains to be determined, however, if interfering with TYK2 expression and/or downstream targets in vivo will impair MPNST initiation and/or maintenance.

### Oestrogen signalling

The development of cNFs in NF1 patients coincides with puberty. Additionally, as discussed by Brosseau et al.^6^, sex hormones have been proposed to influence the growth of tumours in NF1 patients. However, direct evidence is still lacking. The results of a study in an autologous mouse model suggest that pregnancy might favour the development of cNF,^87^ while OPG-induced retinal ganglion loss and retinal nerve fibre layer thinning in mice were reported to depend on the presence of ovaries.^88^ In humans, female NF1 patients are reported to be at a higher risk than the general population of developing breast cancer.^89^ Results from several studies point to a correlation between the *NF1* and oestrogen receptor status in multiple model systems. Oestrogen-receptor-positive breast tumours harbouring a sporadic *NF1* mutation have a poor outcome,^90^ and depletion of oestrogen through ovariectomy in a *NF1*-loss-driven rat model of breast cancer leads to tumour regression.^91^ Breast cancer patients with *NF1* sporadic mutations treated with the oestrogen-receptor antagonist fulvestrant showed a good outcome,^92^ suggesting the potential use of fulvestrant in the context of NF1 patients. Interestingly, it has been shown that neurofibromin directly regulates the oestrogen receptor.^93^ Thus, further elucidating how neurofibromin modulates oestrogen signalling and vice versa might shed light on the role of sex hormones in regulating tumour growth in NF1 patients, and ultimately provide a rationale for repurposing agents that target oestrogen in order to inhibit neurofibroma development.

### Nerve cells

As demonstrated by Liao et al.^94^, the nerve microenvironment plays a key role in initiating neurofibroma formation but the identity of the factors responsible for this process are largely unknown. Neuregulin-1, a Schwann cell growth factor secreted by nerve cells,^95^ binds to its receptors—EGFR and HER2—on Schwann cells to mediate cell growth and migration; notably, overexpression of either neuregulin-1^96^ or EGFR^27^ is sufficient to drive neurofibroma and MPNST tumorigenesis in mice.^27^ Therefore, a potential therapeutic strategy might be to target this neuregulin-1–EGFR axis,^97^ perhaps by repurposing the breast cancer drug trastuzumab.

### Macrophages

pNF-associated macrophages have become a focus in the study of the neurofibroma tumour microenvironment. Indeed microglia were found to be critical to glioma development^98^ and macrophages are abundant in neurofibromas and MPNSTs.^99,100^ These macrophages are recruited by macrophage colony-stimulating factor (M-CSF).^100^ Pharmaceutical inhibition of macrophages has been shown to slow down pNF progression.^100^ Therefore, in addition to tumorous glial cells, these microenvironmental macrophages are also potential therapeutic targets for neurofibroma and glioma.

In the context of OPG, Daginakatte et al.^17,18^ have investigated the role of microglia pharmacologically, using a hyaluronidase inhibitor, and genetically, using established mouse models. Their results indicate that microglia promote the growth of OPG and therefore suggest that the development of strategies targeting stromal cells such as microglia might be pursued.^17,18,98^ This same research group discovered that the expression of numerous cytokines, chemokines and their receptors is altered in microglia in the context of OPG. Indeed, the chemokine ligand CX3CL1 is expressed at abundant levels in the optic nerve and retina, and the expression of CX3CR1 in microglia has been shown to be required for optic glioma formation in vivo.^19^ Monoallelic inactivation of *Cx3cr1* is sufficient to significantly reduce the growth of OPG,^19^ but the use of available small molecule inhibitors against CX3CR1 has so far not been reported. *Ccl5* is also highly expressed in microglia and a neutralising antibody against this chemokine reduced OPG growth and improved retinal dysfunction in vivo in mice;^20^ however, antagonising the CCL5 receptors CCR1 and CCR5, which are expressed on the surface of astrocytes, yielded conflicting results as it had no effect on OPG growth in a mouse model of NF1.^20^ Therefore, the mechanism by which CCL5 exerts its pro-tumorigenic role in the context of OPG remains to be demonstrated.

The absolute role of macrophages in tumorigenesis in the NF1 context is not clear despite their abundant presence in pNF and even more so in MPNSTs in both mice and humans.^100^ Inhibiting the receptor for macrophage M-CSF using PLX3397 successfully decreased macrophage density and promoted tumour regression in a fraction of the mice tested, but only once pNFs had been established. Earlier treatment, prior to neurofibroma formation, increased tumour volume compared with controls, indicating that macrophages are likely to play a defensive role during tumour development but a permissive role once tumours are established.^100^ However, PLX3397 does not selectively inhibit macrophage function: it also inhibits the kinase receptor c-kit, an important positive modulator of neurofibroma growth, on mast cells.^32^ Interestingly, the STAT3 pathway is also hyperactivated in neurofibroma-associated macrophages as well as in neoplastic Schwann cells.^101^ Therefore, the identity of which cells and pathways within the tumour that contribute to the reduced growth of pNFs in response to PLX3397 is unclear. To characterise the neurofibroma-associated macrophages, Choi et al. performed high-throughput gene expression analysis, which confirmed the high expression of *ccl5* in these cells, reinforcing the need to better understand its downstream effectors.^102^ An elevated macrophage number in neurofibroma and nerve requires the express the *Cxcl10* chemokine receptor *Cxcr3* and, notably, *Dhh*-Cre; *Nf1*^f/f^ mice failed to develop neurofibromas in a *Cxcr3*-null background,^66^ suggesting a critical role for the *Cxcl10*–*Cxcr3* chemokine axis in macrophage recruitment for neurofibroma formation. Interestingly, Choi et al.^102^ also discovered a switch from interferon (IFN) type I to type II signalling during the development of neurofibromas, which is consistent with the subsequent modulation of downstream targets of IFN-γ such as colony-stimulating factor (CSF)1.^100,103^ Remarkably, pegylated IFN-α2b significantly slowed down neurofibroma growth in some NF1 patients, warranting further investigation.^104^

In summary, there is a need to better assess the definitive contribution of macrophages to the formation of neurofibromas and MPNSTs in vivo and to pursue the development of strategies that specifically target these cells in future clinical investigation.

### The NF1^+/−^ tumour microenvironment

On average, patients with hereditary cancer syndromes develop tumours at a much younger age than the general population, typically due to a predisposing mutation in a tumour-suppressor gene. Importantly, in addition to the intrinsic tumorigenic drive generated by the biallelic inactivation of this tumour-suppressor gene in the neoplastic cells, it has been suggested that a heterozygous tumour-suppressor microenvironment also modulates tumour progression.^105,106^ In the context of neurofibromatosis, mast cells^32^ and nerve cells^94^ have been shown to accelerate neurofibroma development in vivo. Mast-cell infiltration has been recognised as a histological feature for neurofibroma,^10^ as a high density of mast cells is uncommon in most other tumour tissues. Our recent study showed that pNF-associated mast cells are recruited by tumorous Schwann cell-derived stem cell factor; however, removal of this factor source only slightly affects neurofibroma progression.^11^ This observation suggests that either mast cells might not play an essential role in sustaining pNF tumorigenesis or other immune cells in the heterozygous tumour microenvironment compensate for the loss of mast cell function in this specific murine model. In fact, the roles of macrophages and T cells in promoting NF1-related tumour development have also been shown.^66,100^ Taken together, the coexistence of multiple types of immune cell suggests a complex inflammatory microenvironment in neurofibroma, which could be associated with nerve injury^99^ to promote tumorigenesis. These studies highlight a positive influence of the microenvironment on tumour formation. By contrast, however, Brosseau *et al*. demonstrated that the *Nf1*^+/–^ microenvironment can impair malignant progression.^107^ This result is consistent with the long-standing and puzzling clinical observation of the low malignant potential of the characteristic benign lesions of NF1 patients (e.g. iris hamartomas, café-au-lait macules, cNF). Several mechanisms might account for the impaired malignant progression. One possibility is that heterozygous tumour-suppressor genes might lead to immune cell hyperproliferation and function and, thus, increased immune surveillance, to prevent cancer development.^107^ Consistent with this view, it has been reported that *Nf1*^+/−^ T cells trigger an enhanced immune reaction^107^ and that *Nf1*^+/−^ natural killer cells have a superior protective effect than their wild-type counterparts.^108^ This scenario could have serious clinical implications for organ transplant recipients who coincidentally have NF1, as these patients would have a drastically increased likelihood of developing malignant tumours on treatment with immunosuppressive drugs to prevent transplant rejection. As proof of principle, an association between some autoimmune diseases and NF1 has been reported,^109^ most likely as a result of enhanced immune function due to *NF1* heterozygosity. Initially, this hypothetical model seems to contrast with reports from Dodd et al.^110^ and Hirbe et al.^111^ that a *Nf1*^+/–^ microenvironment accelerates MPNST formation. However, the findings can be reconciled by taking into account the fact that the mouse models used by Dodd et al.^110^ and Hirbe et al.^111^ do not recapitulate the physiological stepwise progression from benign to malignant tumour in human NF1 patients, as is the case in Brosseau et al.^107^. Importantly, this novel working model suggests that identifying the cell types and signalling pathways that underlie the capacity of the microenvironment to suppress malignant progression could open the door to novel treatments for cancer beyond NF1.

### NF1 gene therapy

Research in other monogenic diseases such as muscular dystrophy indicates that even a partial functional rescue of the mutated protein confers patient clinical benefit,^112^ but such a demonstration has so far only been carried out in vitro for NF1.^113^ This is due to the fact that *NF1* is one of the largest genes and its manipulation into expression vectors has proved very challenging. However, given the 2019 report of the successful adeno-associated viral transduction of truncated forms of *NF1* into human cells,^114^ the cloning of a full-length *Nf1* cDNA that is able to modulate Ras signalling,^115^ and opening of a proposal for a proof-of-concept of *NF1* gene therapy by the Children’s Tumor Foundation and the Gilbert Foundation, the NF1 community is optimistic about novel therapeutics aimed at restoring the function of neurofibromin to correct the disease at its root in order to ultimately reduce the tumour burden in NF1 patients.

## Conclusions

NF1 is a hereditary tumour syndrome with poor genotype–phenotype correlation and, hence, unpredictable outcome. Although earlier work has focused on interfering with signalling pathways upstream and downstream of Ras (Fig. 1), current clinical trials are evaluating the efficacy of BRD4 inhibitors and immune checkpoint blockers (Fig. 2). So far, the best results have been obtained by inhibiting MEK (downstream of Ras), but these results are currently limited to pNF and low-grade glioma. Future work is aimed at deciphering the contribution of the Hippo pathway, the JAK/STAT pathway and oestrogen signalling, as well as interfering with cells in the microenvironment that modulate tumour progression, such as nerve cells, T cells, macrophages and *Nf1*^+/–^ stromal cells (Fig. 3).

Combination therapies targeting both neoplastic cells (Schwann cells) and cells of the tumour microenvironment might be more effective than drugs targeting only the tumour cells. Indeed, the *NF1* heterozygous cells of the microenvironment modulate the growth of pNFs,^116^ OPGs^16^ and MPNSTs.^107^ As the tumour mass can contain up to 50% collagen,^48^ strategies aimed at targeting the cells that synthesise collagen and extracellular matrix might be required to shrink the bulk of the tumour, in addition to directly targeting the neoplastic cells. Therefore, further elucidating of the molecular interactions between Schwann cells and their tumour microenvironment will provide new tools and knowledge to develop more effective treatments for one of the most debilitating human genetic disorders in the world today.

### Review criteria

Published research papers, clinical trials, and literature reviews were selected using different keywords (e.g., NF1, neurofibroma, malignant peripheral nerve sheath tumour, optic glioma) and database sources (e.g., PubMed, Google Scholar, ISI Proceedings, Journal storage [JSTOR] Search, Medline, Scopus, Web of Science, Clinicaltrials.gov).

## Data Availability

Not applicable.
